# In situ monitoring reveals cellular environmental instabilities in human pluripotent stem cell culture

**DOI:** 10.1038/s42003-022-03065-w

**Published:** 2022-02-08

**Authors:** Shannon G. Klein, Samhan M. Alsolami, Silvia Arossa, Gerardo Ramos-Mandujano, Anieka J. Parry, Alexandra Steckbauer, Carlos M. Duarte, Mo Li

**Affiliations:** 1 Red Sea Research Center (RSRC) and Computational Bioscience Research Center (CBRC), King Abdullah University of Science and Technology, Thuwal, 23955 Saudi Arabia; 2 Biological and Environmental Science and Engineering Division (BESE), King Abdullah University of Science and Technology, Thuwal, 23955 Saudi Arabia

**Keywords:** Stem-cell biotechnology, Embryonic stem cells

## Abstract

Mammalian cell cultures are a keystone resource in biomedical research, but the results of published experiments often suffer from reproducibility challenges. This has led to a focus on the influence of cell culture conditions on cellular responses and reproducibility of experimental findings. Here, we perform frequent in situ monitoring of dissolved O_2_ and CO_2_ with optical sensor spots and contemporaneous evaluation of cell proliferation and medium pH in standard batch cultures of three widely used human somatic and pluripotent stem cell lines. We collate data from the literature to demonstrate that standard cell cultures consistently exhibit environmental instability, indicating that this may be a pervasive issue affecting experimental findings. Our results show that in vitro cell cultures consistently undergo large departures of environmental parameters during standard batch culture. These findings should catalyze further efforts to increase the relevance of experimental results to the in vivo physiology and enhance reproducibility.

## Abstract (web summary)

Luminescence-based gas measurements on human cell cultures show deviations from physiological conditions, highlighting the need for a careful consideration of experimental results obtained with these standard cell lines.

## Introduction

The degree to which cell culture conditions mimic the in vivo environment and recapitulate the natural behavior and function of cells plays a critical role in the biological relevance of the observations made. One of the most striking discrepancies between routine in vitro mammalian cell cultures and the in vivo environment is the lack of regulatory systems that finely tune levels of oxygenation and acid-base chemistry in the mammalian body to maintain homeostasis^1^. In the mammalian body, there are numerous regulatory systems (e.g., vasodilation, vasoconstriction, changes in respiration rates, and vascular remodeling^2,3^) that maintain levels of dissolved gases and acid-base chemistry within the finite limits that sustain optimal cell function. In contrast, pH levels in routine cell cultures are governed by the equilibrium between media formulations (with a finite buffering capacity) and the CO_2_-enriched atmosphere (usually adjusted to 5 or 10% CO_2_), whereas levels of dissolved gases are governed by surface/atmosphere equilibration. However, cells growing in culture consume O_2_ and release CO_2_ through cellular metabolism. Hence, as cells grow, the culture medium acidifies and deoxygenates to create conditions potentially inconsistent with the levels and stability of the in vivo environment^4^, although the extent of these changes depends on numerous factors, including for example, cell density, the rate of cellular respiration, and the rate of diffusive flux at the air-liquid interface^1^. Cells have evolved sophisticated pathways to sense and respond to minute changes in the in vivo microenvironment^5–9^ and presumably, the same mechanisms govern cellular behaviors in cell culture. However, the environmental changes in routine cell culture are poorly understood even for the staple cell lines used in biomedical research.

It is well established that a broad range of cellular responses are affected by environmental conditions. For instance, departures in pH from the optimal values can change cancer cell behavior, including proliferation, metastasis, metabolic adaptation, and tumorigenesis by altering the structure and function of selective pH-sensitive proteins^10,11^. On the other hand, a 0.5-unit decrease of pH in the microenvironment of human mesenchymal stem cells was shown to adversely affect the osteogenic differentiation in osteoprogenitor cells^12^. Higher CO_2_ (and reduced pH) levels have also been shown to accelerate the differentiation of human preadipocytes in culture, providing mechanistic clues to an interaction between obstructive sleep apnea and obesity hyperventilation syndrome^13^. Fundamental properties of cells in culture, including the lifespan of in vitro cultures^14^ and metabolism itself^15^, show a dependence on O_2_. Reductions in O_2_ also activate the hypoxia-inducible factor (HIF) pathway, which regulates the expression of most genes involved in cellular adaptation to varying O_2_ levels^16–18^ throughout the human body. In the case of tissue stem cells, elevated O_2_ tensions can promote exit of quiescence in hematopoietic stem cells and senescence in mesenchymal stem cells^19,20^, whereas stem cell derivatives, such as 3D organoids, depend on ample oxygenation to proliferate in culture^21–23^. The significance of culturing environments may be especially vital for pluripotent stem cells (PSCs), which are well known to sense and respond to their physical^24–26^ and chemical environment^27–30^. In theory, PSCs possess a unique ability to generate all cell types in the human body^31^ and hence, are a primary driver behind the field of regenerative medicine. Despite this potential, in vitro cultures remain limited in their ability to reliably mimic physiologically relevant processes^32^ to consistently produce PSCs and their derivatives (e.g., cardiomyocytes and blood cells) for use in clinical applications^33,34^.

Despite the manifold effects of pH, CO_2_, and O_2_ on cultured cells and scope for substantial environmental instability in routine mammalian cell cultures, two recent studies assessing the published literature showed surprising neglect for the reporting of critical parameters affecting cell culture environments^1,4^. This lack of reporting may be underscored by widespread misconceptions that cell medium formulations provide enough buffering capacity to offset metabolic acid production and that surface/atmosphere O_2_ exchange suffices to meet the metabolic demands of the culture. A popular medium formulation (e.g., RPMI-1640) may be chosen—mainly based on its ability to support cell proliferation – to grow many types of cells that differ greatly in metabolic activity (e.g., lymphoblastoid cells vs. chronic myelogenous leukemia cells). Surprisingly, there is scant understanding of cell type-specific environmental changes originated from the interaction between cellular metabolic activities and medium buffering capacity and gas equilibration. Although the potential issue of environmental disturbances in standard cell cultures was identified over 50 years ago^35^, we still lack a comprehensive assessment of routine cell culture environments. Indeed, existing characterizations of cell culture environments are presently limited to few cell types and parameters^35–40^, which limits our ability to identify potential issues and implement protocols and standards to correct them. Such a gap in the knowledge of the changes in cell culture environment is in stark contrast to the level of attention given to other biological measurements, often at single-cell and/or single-molecule resolution. Cells are evolved to respond to the changing environment, and therefore any precise measurements of gene or metabolic activities may be influenced by the culture environment. Thus, critical analyses of cell culture environments are urgently needed to improve the interpretability of the compendium of omics data from cultured cells.

There are several emerging technologies capable of monitoring in vitro cell culture environments that range in complexity and affordability. They range from luminescence-based optical sensors^41^ and amperometry sensor chips^42^ to advanced bioreactor culture systems involving high-frequency sensor monitoring^43^. There are also ready-to-use flasks pre-equipped with integrated sets of autoclavable sensors that measure the desired cell culture variables (i.e., pH and dO_2_)^44^. Luminescence-based optical sensors provide an especially powerful tool to characterize levels of dissolved gases in situ in real-time because they can be installed directly on the growth substrate, are highly sensitive, and do not consume dissolved gases or generate toxic byproducts in their sensing process. Here, we integrated optical O_2_ and CO_2_ sensors within the cell culture environment for three model cell lines (human embryonic stem cells (H1 hESC), erythroleukemia cells (K562), and lymphoblastoid cells (GM12878)) widely used in biomedical studies using routine batch culturing procedures. Using real-time measurements of dissolved O_2_ and CO_2_ immediate to the cells, and concomitant measurements of medium pH and cell density, we show that cells experience substantial drifts from the setpoint values in all environmental parameters tested during routine in vitro cultures. Different cell types modified the environment (pH, dCO_2_, and dO_2_) with different dynamics, likely reflecting their different metabolic activity. Accordingly, extracellular lactate measurements were performed and correlated with changes in pH in all cell lines examined. We then conduct an extensive search of the published literature to collate available data of environmental parameters measured during routine cell cultures to show that in vitro cell cultures consistently undergo large departures of environmental parameters, but the extent and nature of variations observed depend on the cell types and culturing protocols used.

## Results

**Fig. 1:**
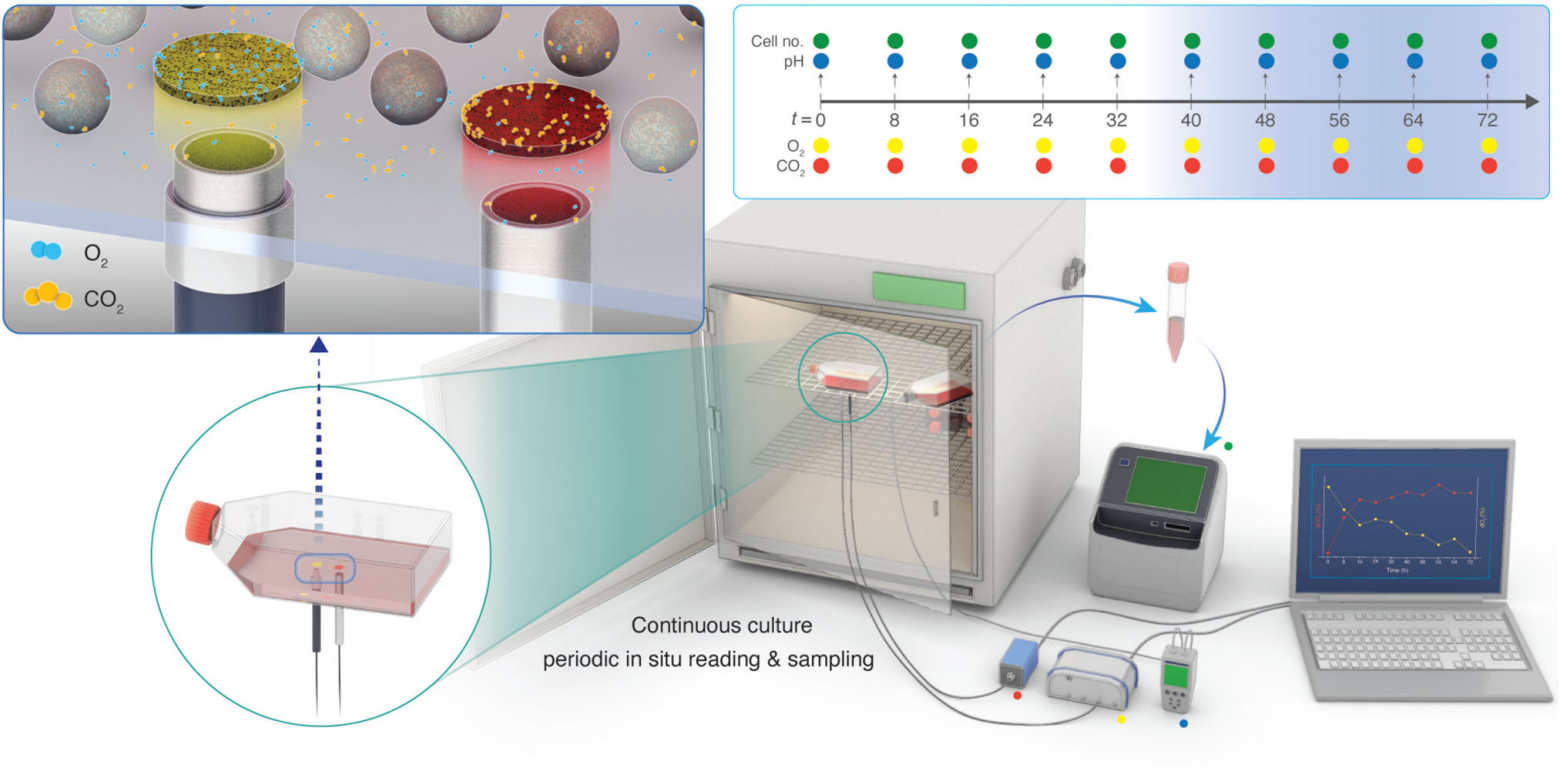
Non-invasive monitoring of dissolved gases in routine human cell cultures using luminescence-based optical sensors. (Top left inset) simplified schematic depicting a macroscopic view of the dO_2_ (left) and dCO_2_ (right) sensor spots affixed to the growth substrate (inside of the culture vessel). The fiber-optic probes transfer light at a specified wavelength from outside of the vessel to excite the sensor spots, which then emit a fluorescent signal. These signals are equated to concentration values for dO_2_ and dCO_2_. For each cell line assessed, levels of dO_2_ and dCO_2_ were non-invasively monitored in the culture medium of three identical flasks using luminescence-based optical sensor spots every 8 h. In parallel, three culture flasks were sacrificially sampled for pH measurements and live-cell counts, yielding a time course of pH, dO_2_, dCO_2_, and live cell counts. (Top right inset) timeline of sampling regime showing the timing of pH (blue dots), dO_2_ (yellow) dots, dCO_2_ (red dots), and live cell counts (green dots). *n* = 3 measurements (biological replicates) for each variable at each 8 h time-point.

We undertook several measures to control the factors that could introduce variability in the measurement of environmental parameters in the trials (see Methods). Briefly, the passage number of the H1 hESC, K562, and GM12878 cells, the lot of the media, the lot of the serum, and the initial culture state (e.g., viability, density, etc.) were kept the same throughout all experiments. We used luminescence-based optical sensor spots to characterize levels of dissolved gases (dCO_2_ and dO_2_) in the culture environment. Although optical sensor spots have been previously used to characterize levels of dO_2_ in the culture environment^40,44–46^, this study extends the use of optical sensors to evaluate dCO_2_ in the cellular microenvironments during routine in vitro cultures. For each cell line, the optical dCO_2_ and dO_2_ spots were affixed to the cell growth surface of three replicate T-25 flasks (for H1 hESC) or T-75 flasks (for K562 and GM12878) using silicone glue, in accordance with manufacturers’ recommendations (see Methods, Fig. 1). Two fiber optic probes, attached to a compact fiber optic CO_2_ meter and a fiber optic O_2_ meter, were positioned directly below each sensor spot (on the outside of the vessel) to excite fluorescence, yielding non-invasive measurements of dCO_2_ and dO_2_ at the growth substrate (Fig. 1). Specifically, the fluorescence measurements were equated to levels of dCO_2_ and dO_2_ and compensated for temperature and pressure (see Methods). dCO_2_ and dO_2_ were repeatedly measured at 8 h intervals using three identical replicate flasks. Three additional replicate flasks were sacrificed at each time point for pH measurements and cell counts (Fig. 1). All environmental parameters were measured promptly after the incubator door was opened to minimize the influence of atmospheric and temperature fluctuations on the medium environments.

**Fig. 2:**
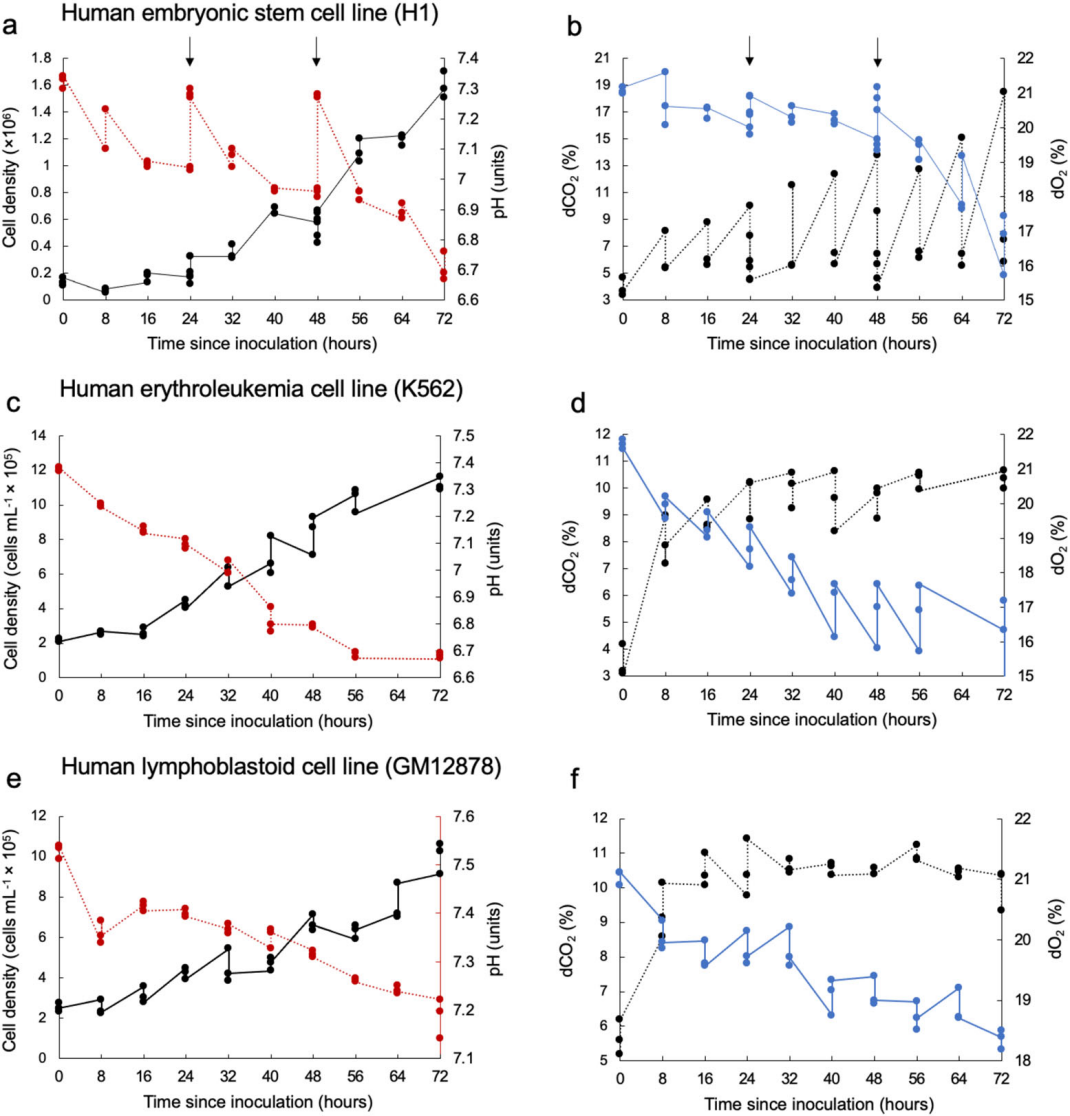
Measured environmental conditions in human cell cultures. **a** Time course of cell density (black data points) and medium pH (red data points) in cultures of human embryonic stem cells (H1 hESC, seeding density 1.8 × 10^5^ in T25 flasks with a growth area of 25 cm^2^ and working volume of 5 mL). **b** Time course of dCO_2_ (black data points) and dO_2_ (blue data points) in cultures of H1 hESC. Medium buffered with 5% CO_2_/22 mM HCO^3−^ plus 15 mM HEPES. Arrows in panels (**a**) and (**b**) represent the timing of 24 h medium exchanges for the H1 hESC cell line. **c** Time course of cell density (black data points) and medium pH (red data points) in cultures of human erythroleukemia cells (K562, seeding density 2.0 × 10^5^  mL^−1^ in T75 flasks with a growth area of 75cm^2^ and working volume of 20 mL). **d** Time course of dCO_2_ (black data points) and dO_2_ (blue data points) in cultures of K562. Medium buffered with 5% CO_2_/22 mM HCO^3−^. **e** Time course of cell density (black data points) and medium pH (red data points) in cultures of human lymphoblastoid cells (GM12878, seeding density 2.0 × 10^5^ mL^−1^ in T75 flasks with a growth area of 75 cm^2^ and working volume of 20 mL). **f** Time course of dCO_2_ (black data points) and dO_2_ (blue data points) in cultures of GM12878. Medium buffered with 5% CO_2_/22 mM HCO^3−^. Measurements conducted for three culture flasks per cell line (three biological replicates each). Data points at each time point represent data for the three biological replicates.

**Fig. 3:**
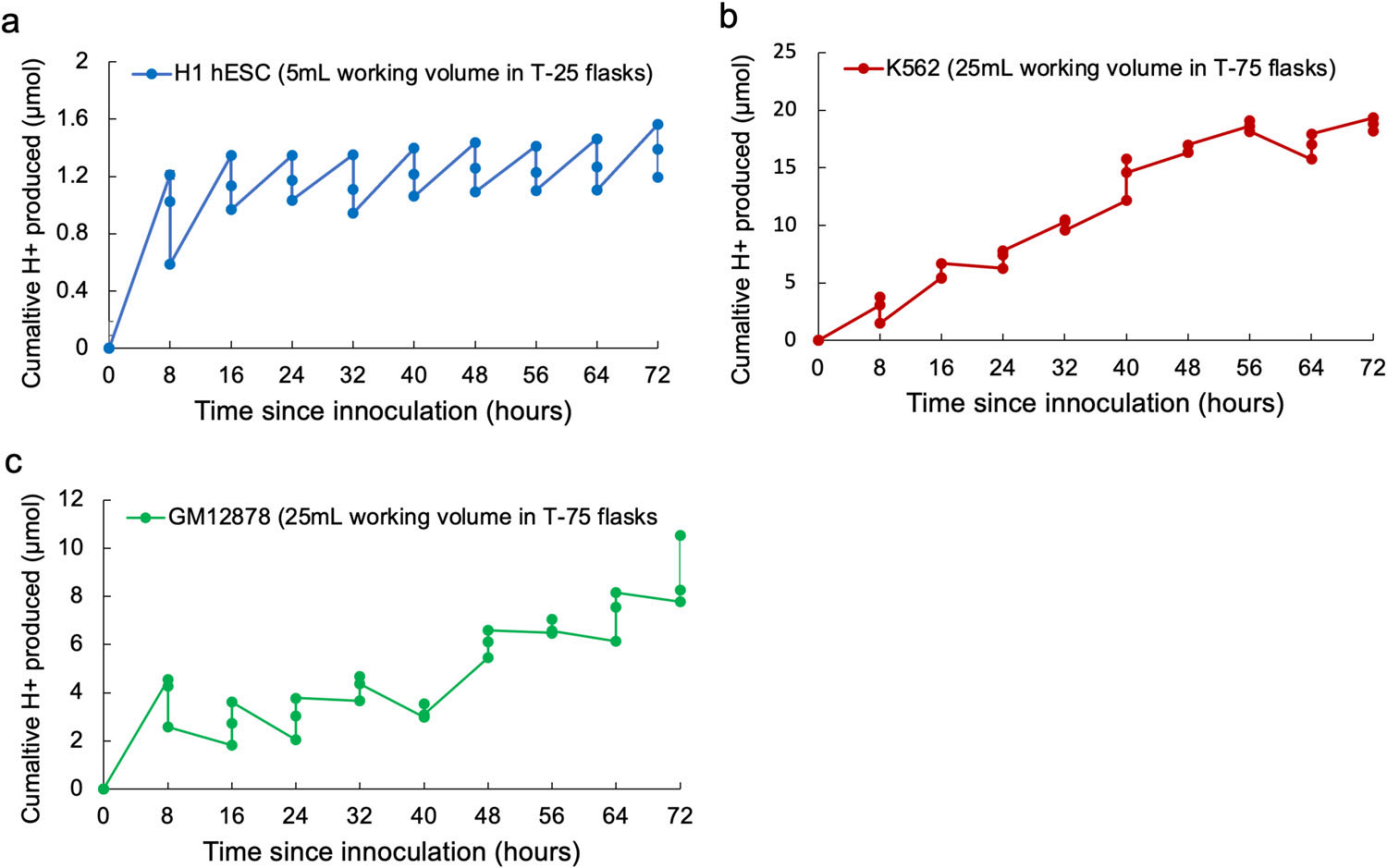
Cumulative H^+^ production in human cell cultures. Time course of cumulative H^+^ production in the (**a**) H1 hESC, (**b**) K562, and (**c**) GM12878 cell lines, which were calculated using Eq. (1) (see Methods) following ref. ^47^. The legend specifies differences in working volume and flask type among the cell lines. Measurements conducted for three culture flasks per cell line (three biological replicates each). Data points at each time point represent data for the three biological replicates.

**Fig. 4:**
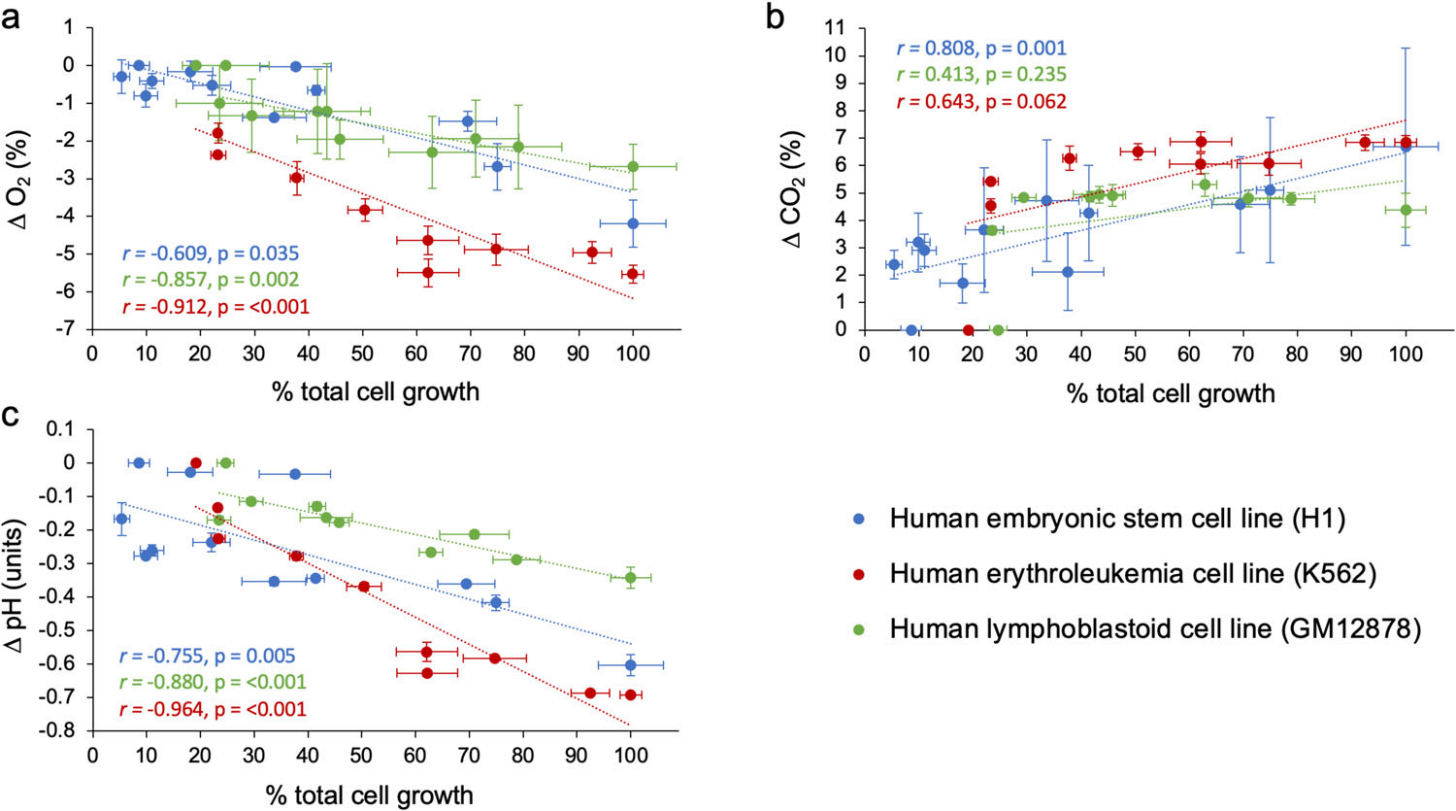
A comparison of relationships between changes in environmental parameters and cell growth in cultures of H1 hESC, K562, and GM12878. Delta (∆) values for (**a**) dO_2_, (**b**) dCO_2_, and (**c**) pH represent the mean difference between levels measured at 8 h intervals minus measurements taken at time zero. Percent cell growth was calculated based on the assumption that cell densities at 72 h represent 100% cell growth and used to facilitate comparison among the cell line cultures despite differences in cell density units. Data points represent mean values across the three replicate flasks ±1 standard error for each cell line. Insets show the Pearson correlation coefficient (*r*) and *p*-value for each correlation model (cf. Supplementary Table S1 for full statistical results).

The effect of metabolic CO_2_ production in decreasing medium pH was evident across all three cell lines examined and co-occurred with increases in cell density over the culture period (Fig. 2b, d, and f, Supplementary Table S1). However, the extent and rate of acidification differed among the three human cell lines (H1 hESC, K562, and GM12878), with the largest pH declines observed in the H1 hESC and K562 cell lines after 72 h of culture, despite daily medium exchanges in the H1 hESC culturing procedure (Fig. 2a, c, e). In the cultures examined here, reductions in pH ranged from 0.32 pH units in the GM12878 cell line to 0.7 pH units in the K562 cell line (Fig. 2a, c, e), exposing cells to extracellular pH variations inconsistent with conditions in vivo. Consistent reductions in pH across the three cell lines nearly perfectly correlated (R^2^ ~ 0.99) with extracellular lactate accumulation over the 72 h of culture (Supplementary Fig. S1b–d). Quantifying the extent to which cellular acid production (via CO_2_ hydration and/or lactic acid) underpins medium acidification requires consideration of media pH buffering capacity. For this, we measured the buffering capacity of the two media used (E8 medium: H1 hESC and RPMI-1640: K562 and GM12878) and calculated cumulative H^+^ (C_H+_) production to resolve cellular acid production across the three cell lines (as described in previously^47^, and also see Methods). Although the time courses of C_H+_ (Fig. 3) show that H1 hESC had low rates of H^+^ production relative to the other cell lines, the lower buffering capacity of the E8 medium (relative to RPMI-1640, Supplementary Fig. S2) resulted in substantial pH reductions after 72 h culture (Figs. 2a, c, e, and 4c, Supplementary Table S1). Conversely, K562 had strikingly high rates of H^+^ production, equating to C_H+_ production 2–3 times higher than the other two cell lines (respectively) after 72 h of culture, which resulted in the largest degree of medium acidification (Figs. 2a, c, e and 4c).

Similar to our observations about pH, the effect of cellular metabolism on dissolved gases was apparent. Across the cell lines examined, dO_2_ levels consistently reduced as cell densities increased over the 72 h culture (Fig. 2b, d, f, Supplementary Fig. S3). We also observed concomitant accumulations of extracellular lactate, a known byproduct of glycolysis, over the same timeframe (Supplementary Fig. S1a). In the H1 hESC and K562 cell cultures, dO_2_ levels at the growth substrate position fell below the expected concentration of dO_2_ in an incubator under a controlled atmosphere of 5% CO_2_ in air (20.9 to 18.6% see^43^), likely resulting in meaningful alterations to oxygen delivery per cell (Figs. 2b, d and  4a). Average levels of dCO_2_ far surpassed the expected CO_2_ level of 5% after 72 h of culture (Figs. 2b, d, f and 4b).

**Fig. 5:**
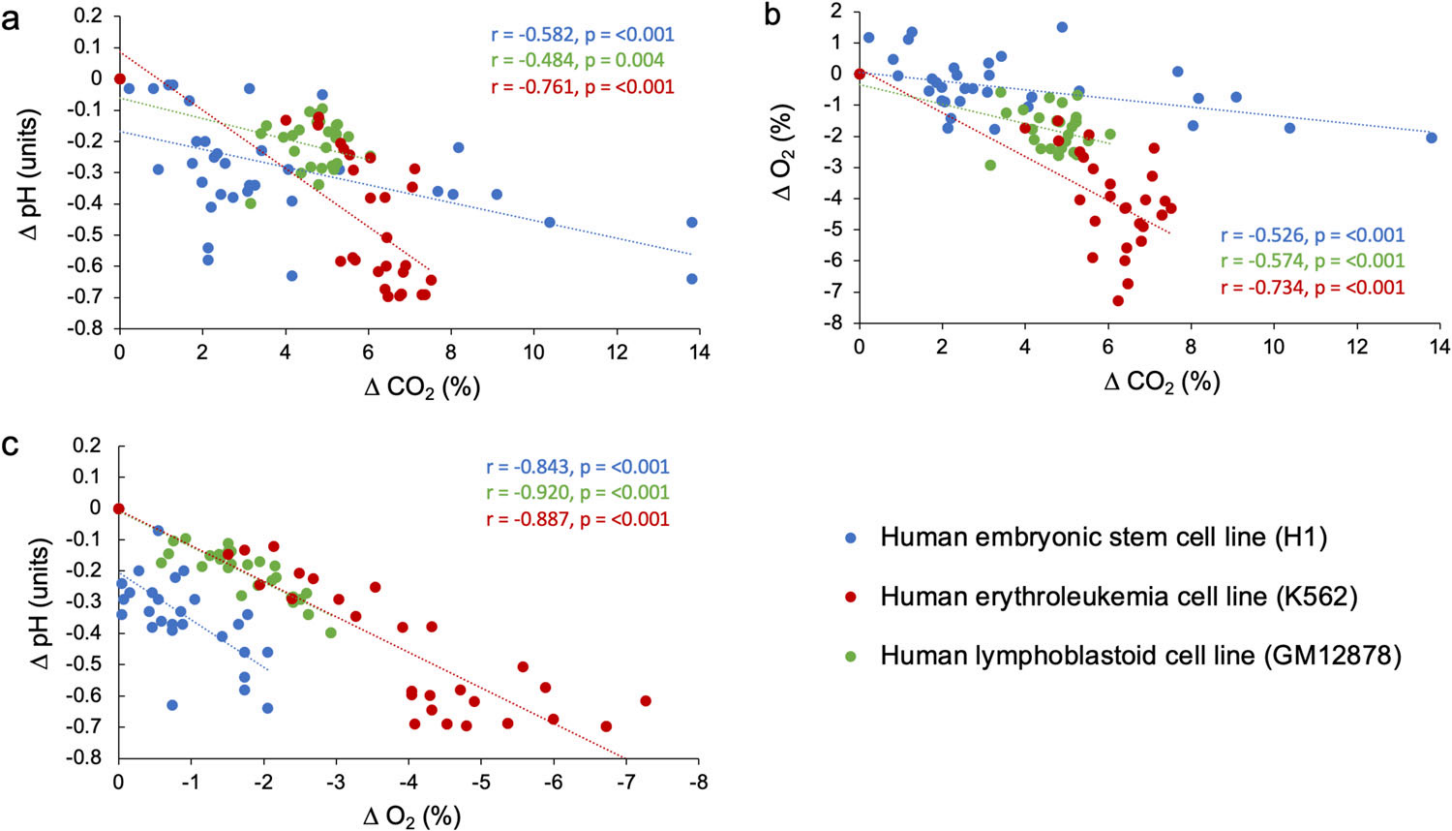
Relationships between changes in dissolved gases and medium acidification in cultures of H1 hESC, K562, and GM12878. Delta (∆) values for the environmental variables (pH, dO_2_, and dCO_2_) represent the mean difference between levels measured at 8 h intervals minus measurements taken at time zero. **a** The relationship between reductions in pH and increases in dCO_2_ in cell culture media. **b** The relationship between reductions in dO_2_ and increases in dCO_2_ in cell culture media. **c** The relationship between reductions in pH and dO_2_ in cell culture media. Data points represent individual measurements taken from three culture flasks per cell line. Insets show the Pearson correlation coefficient (*r*) and *p*-value for each correlation model (cf. Supplementary Table S2 for full statistical results).

The relationships between changes in dissolved gases (O_2_ and CO_2_) and medium acidification (with consideration of medium buffering capacity) can provide insights into the mechanisms involved. Increases in dCO_2_ were significantly correlated with declines in pH and dO_2_ across the cell line cultures (Fig. 5a, b, Supplementary Table S2), suggesting aerobic metabolism as a driver of both pH declines and deoxygenation in the culture media. However, the magnitude (or steepness) of the changes differed among the cell types investigated. For instance, the K562 cell line exhibited the largest decreases in pH among the cell types, but reached dCO_2_ levels only slightly higher than GM12878, which was also cultured in RPMI-1640 and exhibited the smallest decreases in pH (Fig. 5b, c). These observations align with the C_H+_ time courses shown in Fig. 3, where K562 produced substantially higher quantities of H^+^ than GM12878 and H1 hESC. Differences in cell growth rates are a plausible explanation for these differences. Indeed, the GM12878 cell line had ~40% less cells than the K562 cell line after 72 h of culture following the same culturing procedure (Fig. 2a, e). Another explanation for these differences could be in the metabolic plasticity of the cell lines assessed. Particular stem cells (including hESCs) and cancer cells are known to engage more in glycolysis than in oxidative phosphorylation (OXPHOS)^48–50^, leading to the formation of large quantities of lactic acid in the culture medium without substantial consumption of dO_2_. These data suggest that cell lines known to engage in glycolytic metabolism and exhibit high growth rates may be especially prone to substantial pH variations in the in vitro cell culture^37^, through metabolic release of both CO_2_ and lactic acid.

**Fig. 6:**
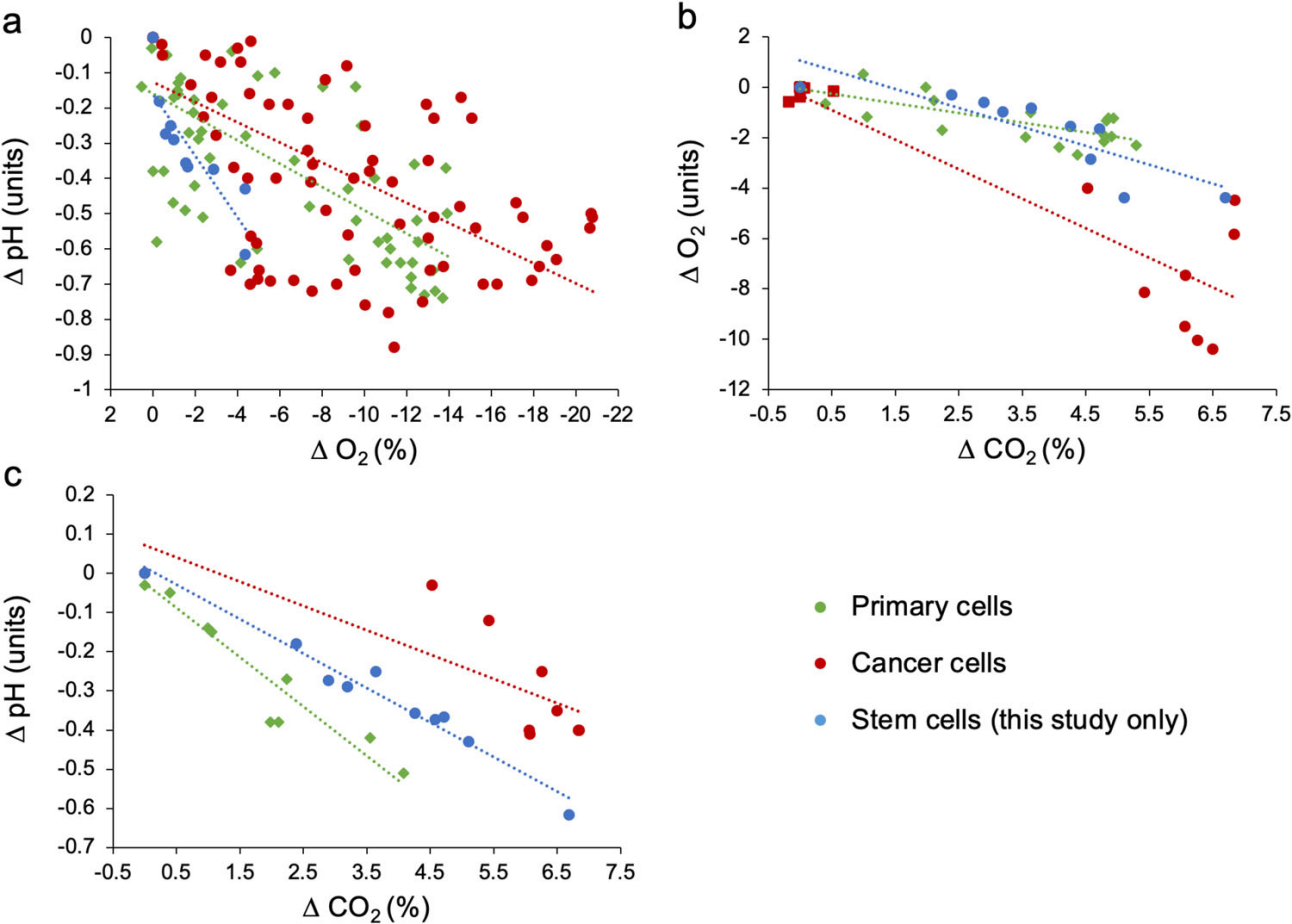
Relationships between changes in dissolved gases and medium acidification in mammalian cell cultures. Delta (∆) values for pH, dO_2_, and dCO_2_ represent the mean difference between levels measured at various time points during culture minus measurements taken at time zero (cf. Supplementary Figs. S3 and S4). **a** The relationship between reductions in pH and increases in dO_2_ in cell culture media. **b** The relationship between reductions in dO_2_ and increases in dCO_2_ in cell culture media. **c** The relationship between reductions in pH and dCO_2_ in cell culture media. Data points represent a combination of mean ∆ values extracted from six published studies (examining nine cell lines) and mean ∆ values obtained from this study. Insets show the Pearson correlation coefficient (*r*) and *p*-value for each correlation model (Supplementary Table S3). Cell lines were categorized as belonging to one of three main categories (primary cells, cancer cells, or stem cells, cf. data availability section for full dataset).

To evaluate the generalizability of the observations made in this study, a publication search (see methods for search strategy) was conducted in February 2020 to obtain data of pH, dO_2_, and dCO_2_ in media of mammalian cell cultures. The resulting dataset delivered 203 measurements of 12 different cell lines from seven published studies, examining primary cells, cancer cells, and stem cells that were published between 1971–2019 and included our own data (presented in Fig. 2). Regardless of cell type (primary vs. cancer vs. stem cells), the role of cellular metabolic activity in acidifying and deoxygenating the culture medium was evident in all cell line cultures examined (Fig. 6a–c). Increases in dCO_2_ were significantly correlated with reductions in pH and dO_2_ across the experiments (Fig. 6a, b, Supplementary Table S3), signifying a role of aerobic metabolism in modifying a broad range of culture environments. Although our study is the only assessment of a stem cell line culture (H1 hESC), the relationship between changes in pH and dO_2_ was steeper in H1 hESC culture than that in cancer and primary cell line cultures (Fig. 6a, Supplementary Table S2). These data further indicate that although the H1 hESC line, and potentially also other stem cell types, can exhibit moderate reductions in dO_2_, they may be vulnerable to extreme degrees of acidification, which would necessitate interventions. Overall, environmental instability is universally observed, and its extent varied among the cell lines investigated and/or the culturing procedures used, as well as the culture incubation period (Supplementary Figs. S4, S5, Supplementary Table S3).

## Discussion

Our findings, consistent with the existing published evidence^35,37–40^, show that departures in pH, dO_2_, and dCO_2_ from physiological conditions exist in almost all of the cultures examined. Overall, the extent of environmental instability varied among the cell culture experiments assessed, highlighting the vital need to monitor environmental conditions in cell cultures, report factors known to directly and indirectly affect culture conditions, and implement steps to ensure physiological conditions. Hence, the potentially widespread assumption that routine cell-culturing procedures are sufficient to maintain environmental stability should be urgently reconsidered. Indeed, as with bespoke media, our results show that commercial media, including those assessed here (E8 and RPMI-1640), do not guard cultured cells against environmental instability even when following the manufacturer’s instructions and field-standard protocols. Additionally, different cell types behave differently when cultured in the same medium, suggesting that cell growth rates and the metabolic profile of the cells are critical factors that determine the environmental stability of the culture.

Physiologically, principal sources of acid include aerobic metabolism, via the hydration of CO_2_ yielding H^+^ ions, and anaerobic glycolysis through the formation of lactic acid^51^. In cell cultures, both reactions can inadvertently reduce medium pH. Although pH levels vary across in vivo tissues, pH levels within individual compartments are highly regulated^52–54^. For instance, pH in mammalian arterial blood is strictly maintained between 7.35 and 7.45 pH units, and reductions (as seen in the cell cultures examined here) beyond this range cause acid-base abnormalities that can lead to mortality^55,56^. In the case of O_2_, the levels under which human cells exist in vivo vary across human tissues (ranging from 1% in the large intestine to 13% in the lung-pulmonary vein) but are substantially lower than those in standard batch culture conditions (ranging from 20.9 to 18.6% O_2_^43^)^15^. Although dO_2_ levels in two of the cell lines examined here (H1 hESC and K562) fell below expected concentrations of dO_2_ in media under a controlled atmosphere of 5% CO_2_ in air (20.9% to 18.6%^15,46^), O_2_ levels in all three cell lines nevertheless represented O_2_ tensions far higher than in vivo conditions for any cell type, even after 72 h of culture. While the manifold effects of pH, O_2_, and CO_2_ on a broad range of cellular responses are widely recognized, published experiments often fail to monitor and report cell culture environments and the factors affecting them^1,4^. Indeed, increases in dCO_2_ alone can considerably affect cellular function^57,58^ because CO_2_ is an important signaling molecule.

PSCs hold great promise in generating all cell types in the human body. Careful controls over the in vitro culture environment are required to reliably produce the intended PSC progeny capable of recapitulating in vivo physiology^59^. Here, we provide a thorough assessment of the routine culture environment for human PSCs, which revealed substantial environmental variations that depart from physiological conditions. pH is a critical culture parameter recognized to affect PSC reprogramming and differentiation through its influence on differential splicing, mitochondrial activity, signaling pathways associated with the cellular plasma membrane, and liquid-liquid phase separation^29,60^. Also, pH changes have been shown to affect chromatin acetylation levels in ESCs, where lowered pH induces histone deacetylation to buffer acid-base disturbances^61^. Dissolved gases, including O_2_, are also known to influence the reprogramming and differentiation of PSCs^62–64^. Hence, the extent of environmental deviations observed here likely affects the maintenance and differentiation of PSCs. How these uncontrolled culture environmental instabilities affect cellular reprogramming^29^ and the heterogeneity of PSC culture^30^ warrants further study. The monitoring and precise control of environmental parameters in stem cell cultures are of great importance if the full potential of PSCs is to be reached.

Although investigations into how environmental instability in routine human cell cultures affects reproducibility are scarce, recent evidence shows that the influence of cellular metabolism on the culture medium environment may be difficult to reproduce and result in non-intuitive outcomes^4,37,38,65^. Solving this issue requires a systematic approach, which should first involve identifying the causes and impacts of variability in culture conditions, for which this study provides an initial insight. The second task will be to ensure the necessary infrastructure required to routinely monitor and, where required, control environmental regimes in cell cultures. As an initial assessment, the protocol developed in the present study (using fluorescence-based optical sensor technology) provides an affordable and non-invasive tool to characterize the environment cells are experiencing during standard batch cultures. Although few studies have addressed this issue to date, wider recommendations for the monitoring, control, and reporting of environmental conditions in cell cultures are already available^1,4,37,66^. For instance, amendments to existing batch culture protocols, involving, for example, reductions in cell density, increases in the frequency of passages, and the adaptation of culture vessels to increase “headspace” (to allow for efficient surface-area equilibration), could constrain environmental drift to within acceptable (physiological) ranges^1,4,66^. There are also commercially available systems–including chemostats, perfusion systems, and advanced bioreactor systems^43,67,68^–that are designed to maintain stable environmental conditions via either the continuous dilution with fresh medium or the automated addition of gases (O_2_ and CO_2_) and acids/bases to maintain set targets^69,70^. The large drift of environmental parameters (pH, dO_2_, and dCO_2_) in cell cultures away from the stability of the in vivo environment is likely to influence experimental findings. Improved procedures to control these parameters provide an opportunity to increase the relevance of experimental results to the in vivo physiology and enhance their reproducibility^1,4,15^.

## Methods

### Cell line culture conditions

Human embryonic stem cells (H1 hESC; Wicell), human lymphoblastoid cells (GM12878 cell line; Coriell Institute), and human chronic myelogenous leukemia cells (K562 cell line; ATCC) were used for the batch culture experiments. All cell lines (H1 hESC, GM12878, and K562) were expanded and cryo-preserved in the vapor phase of a nitrogen (N_2_) tank for long-term storage. The passage number of the GM12878 cell line was maintained after being received from its source and passage 7 was used throughout this experiment. The passage number used for H1 hESC line was passage 38. The H1 hESC line was maintained in commercial E8 medium and both GM127878 cells and K562 cells were maintained in RPMI-1640 medium supplemented with 10% FBS. The cell culture medium for GM12878 and K562 was supplemented with cell-culture grade sodium bicarbonate (NaHCO_3_^−^), to act as a buffer against CO_2_ enrichment, and contained 1% Pen/Strep. Throughout the experiments, the passage number of the H1 hESC, K562, and GM12878 cells, the lot of the media, the lot of the serum, and the initial culture state (e.g., viability, density, etc.) were kept the same. hESCs were cultured on a pre-coated rhlaminin-521 T-25 flasks, and cells were allowed to adhere for 24 h prior to each experiment, and these cells were detached using the TrypLE reagent (Thermo Fisher Scientific) for 5–10 min. The E8 culture medium has both HEPES (15 mM) and sodium bicarbonate (1.743 g L^−1^). We conducted daily medium exchanges for the H1 hESC cell line so as to adhere to standard culturing practices. The batch cell cultures were maintained in HERAcell 150i incubators (Thermo Fisher Scientific) and the internal atmosphere contained 5% CO_2_ in air, and a humidity of ~90%. All cell lines were routinely checked for mycoplasma contamination using the Lonza mycoalert kit, and continuously were monitored closely under an inverted microscope (at 20× magnification) for any sign of microbial contaminations (i.e., bacteria). Additionally, during regular cell culture, GM12878 and K562 cells were kept in a working volume of 20 mL in T-75 flasks in the horizontal position, and cell numbers were held at a density under 800k mL^−1^ to maintain a healthy environment. Also, H1 hESC cell line was kept in a working volume of 5 mL in T-25 flask. Additional information on reagents, equipment, and software is provided in Supplementary Table S4.

### Measurements of dissolved gases, medium pH, and cell density

GM12878 and K562 cells were thawed and given 40 h for post thaw recovery prior to the start of the experiment. The GM12878 and K562 cells were inoculated at 200k mL^−1^ in T-75 flasks with a working volume of 20 mL, whereas H1 hESC cells were seeded at 180k cells (total) in the T-25 flasks. The optical dCO_2_ and dO_2_ spots were affixed to the inner bottom surface of three replicate T-75 flasks as well as the T-25 flasks using silicone glue, in accordance with manufacturers’ recommendations (see Supplementary Table S4). Briefly, all equipment used to install the sensor spots were autoclaved at 120 °C for 20 min to prevent contamination. The flasks were left in darkness until the glue had curated (~18 min) to avoid light-induced damage to the sensor spots and then each flask was sterilized using 50% EtOH, rinsed with DPBS, followed by RPMI-1640 medium. The fluorescence measurements were equated to levels of dCO_2_ and dO_2_ using commercial software (PreSens Measurement Studio 2, PreSens) and compensated for temperature and pressure (see Supplementary Table S4). The dO_2_ sensor spots were calibrated using a two-point calibration (100% air saturation, and 0% O_2_ via the displacement using 99.999% pure N_2_ gas). A custom calibration protocol for the dCO_2_ sensor spots was done by the manufacturer (PreSens) for measurements at 37 °C and expected dCO_2_ levels ranging between 0 and 25%. The dCO_2_ sensor spots were also custom calibrated for the osmolarity (between 279 to 320 mOSM kg^−1^) of the culture media used. We also used culture media (RPMI-1640 and E8 medium) equilibrated to three custom gas mixtures (0% CO_2_ in air, 5% CO_2_ in air, and 15% CO_2_ in air) after each experiment to verify the accuracy of the CO_2_ spots after 72 h of culture.

All flasks were maintained, and measurements recorded, in a HERAcell 150i incubator (Thermo Fisher Scientific) at 37 °C and the incubator atmosphere contained 5% CO_2_ in air, and humidity of ~90%. For each cell line assessed, levels of dO_2_ and dCO_2_ were non-invasively monitored in the culture medium of three identical flasks every 8 h using the optical sensor spots. In parallel, and at each 8 h time-point, three culture flasks were sacrificially sampled for pH measurements and live-cell counts, yielding a time course of pH, dO_2_, dCO_2_, and live cell counts. For the H1 hESC cell line, we measured pH, dO_2_, and dCO_2_ at two additional time points (after the medium exchanges at 24 and 48 h) to capture the impact of these exchanges on the environmental parameters measured (Fig. 1). In the case of the H1 hESC cell line, we included measurements of pH, dO_2_, and dCO_2_ levels taken before and after the medium exchanges in all analyses, including our analyses of correlated changes in the parameters (Figs. 4, 5, and 6, see also Supplementary Tables S1 and S2 for differences in statistical power among cell lines). The manufacture’s calibration for temperature, CO_2_, and O_2_ of the incubator atmosphere was verified 2 weeks prior to the commencement of the experiments using independent meters. The sampled cells were centrifuged (5 min at 250 RPM) and the pellet was resuspended in the appropriate complete medium. Cells were stained using trypan blue (for 3 min), placed in disposable slides chambers, and two counts per sample were performed following manufacturer recommendations (Countess II automated cell counter, Thermo Fisher Scientific).

### Extracellular lactate measurements

An independent experiment was conducted to measure extracellular lactate concentrations produced by the H1 hESC, K562, and GM12878 cell lines. We followed the same protocols and steps as described above so that measurements of each cell lines reflect the same measured conditions. Briefly, we used a microplate reader coulometric kit (Sigma-Aldrich, cat# MAK064) to measure extracellular lactate concentrations at 24, 48, 72 h, using three biological samples with duplicate technical measurements for each reaction. This assay kit determines lactate concentration via an enzymatic assay, with sensitivity levels ranging from 0.001 mM to 10 mM. At each timepoint, aliquots of the supernatant medium were collected and centrifuged at 200 g for 5 min to remove cells and/or debris. The samples were then diluted with the assay buffer provided. Lactate standards supplied were used to construct a standard curve using a standard lactate serial dilution (0.0 mM, 0.04 mM, 0.08 mM, 0.12 mM, 0.16 mM, 0.2 mM, and 0.4 mM). Finally, the excel “trend”, function was implemented to equate the lactate concentration in the unknown samples’ via measured spectrophotometric absorbances of the standards at 570 nm (A_570_). The data generated from this assay are reported in Supplementary Fig. S1a–d.

### Calculations of cumulative acid production and determination of media buffering capacity

We used the time-courses of pH measured in the 72 h batch cultures of H1 hESC, K562, and GM12878 cell lines (Fig. 2a, c, e) and cell-free media (Supplementary Fig. S6, see also Supplementary Fig. S7 for dO_2_ and dCO_2_) to calculate cumulative acid (H^+^) production (C_H+_) in μmol, according to the following equation and following ref: ^47^

1 $${C}_{H+} 	=V\times \left(\mathop{\sum }\limits_{t=0}^{T}{{J}_{H+}}^{cell}-\mathop{\sum }\limits_{t=0}^{T}{{J}_{H+}}^{cell-free}\right)\\ 	=V\times \left(-\mathop{\sum }\limits_{t=0}^{T}\left(\frac{\Delta p{H}^{cell}}{\Delta t}\times \beta \right)+\left(\frac{\Delta p{H}^{cell-free}}{\Delta t}\times \beta \right)\right)$$

where, V is the volume of the culture medium (in µL), *J*_H+_^cell^ is the acid flux calculated for batch cultures containing cells (Fig. 2) and *J*_H+_^cell-free^ is the acid flux calculated for identical batch cultures of cell-free media (Supplementary Fig. S6), *t* is time (in hours), and *β* is the pH buffering capacity of the medium (Supplementary Fig. S2). Delta (Δ)pH represents final—initial values for each time-point. For RPMI-1640 and E8, we measured the *β* using a stepwise addition of 0.5 mM HCl to the culture media and recorded pH at each step. We plotted pH against mM of HCl added (Supplementary Fig. S2) and calculated the inverse of slope, which provides the average *β* in M/ΔpH (determined as 0.00909 and 0.00769 for RPMI-1640 and E8 medium, respectively).

### Published data of environmental parameters measured in routine cell line cultures

In the publication search, we used a combination of keywords in PubMed NCBI^®^ and Google Scholar search engines, which included “cell culture” AND “pH”, “O_2_”, “CO_2_”, “buffering capacity”, “dissolved oxygen”, “dissolved carbon dioxide”, “dissolved gases”. We extracted mean pH, O_2_, and CO_2_ values, as well as the time at which the values were recorded throughout the incubation (reported in hours since inoculation). We used these data to produce ∆ (delta) values that represented the mean difference between levels measured at various time points during culture minus measurements taken at time zero. We collected mean pH, dO_2_, and dCO_2_ values for all cell cultures within each publication, including those of the same cell type with slight differences in media formulations (e.g., higher or lower concentrations of serum or differing concentrations of buffers) to represent the diversity of changes expected in mammalian cell cultures. Where publications reported a time-series of pH, O_2_, and CO_2_ values, we extracted data for incubation times that were common across the publications to facilitate comparison (see Data Availability section). Publications reported CO_2_ and O_2_ concentrations in different units (mmHg, % air saturation, and absolute %) and were converted into units of an absolute percent (%) under the assumption that experiments were conducted at ambient atmospheric pressure (101.325 kPa).

### Statistics and reproducibility

The relationships between changes in dissolved gases and medium pH were analyzed using bivariate *Pearson correlations in SPSS version 27 (cf*. Supplementary Tables S1, S2). Supplementary Figs. 2, 3, 4, and 5 were made in the Microsoft excel software and depict individual data points, except for Fig. 4 that displays *mean*  ±  *1 standard error* of data depicted in Fig. 2. Alpha was set to 0.05 for all analyses and differences were considered significant when *p* < 0.05. The statistical tests used are specified either in the text and/or figure legends, where appropriate.

### Reporting Summary

Further information on research design is available in the Nature Research Reporting Summary linked to this article.

## Supplementary information

Supplementary Information

Reporting Summary

## Data Availability

The dataset containing published data of environmental parameters measured in routine cell line cultures generated and analyzed during the current study is available at 10.5061/dryad.41ns1rnd9^71^.
