# Supplementary material for: In situ monitoring reveals cellular environmental instabilities in human pluripotent stem cell culture
Source: Commun Biol. 2022 Feb 8;5:119. doi: 10.1038/s42003-022-03065-w (PMC8826360; doi:10.1038/s42003-022-03065-w)
Supplement: Supplementary file 1 — Supplementary Information [file 42003_2022_3065_MOESM1_ESM.pdf]

Supplementary Information

In situ monitoring reveals cellular environmental instabilities in human pluripotent stem cell culture

Shannon G. Klein[1,+], Samhan M. Alsolami[2,+], Silvia Arossa[1], Gerardo Ramos Mandujano[2], Anieka J. Parry[1], Alexandra Steckbauer[1], Carlos M. Duarte[1,*], and Mo Li[2,*].

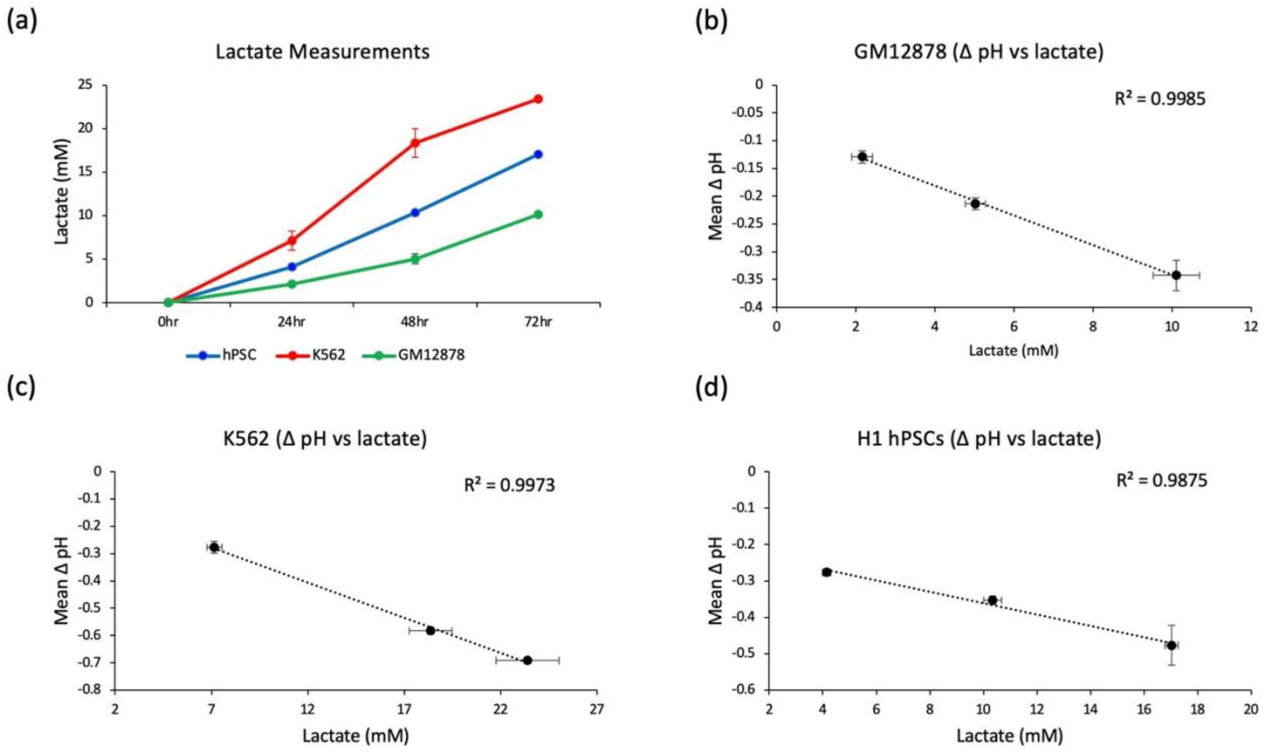

Figure S1. Extracellular lactate measurements over the period of 72 hours for H1 hESC, K562, and GM12878, and the correlation of these data with changes in pH over the same period of time. (a) Lactate microplate reader absorbance measurements for H1 hESC, K562, and GM12878, expressed in mM as referenced to a standard curve (cf. Methods). Measurements were done in duplicates for three biological replicates. (b-d) Lactate measurements correlated with changes of pH over the 72-hour culture period. Data points represent mean ± 1 standard error.

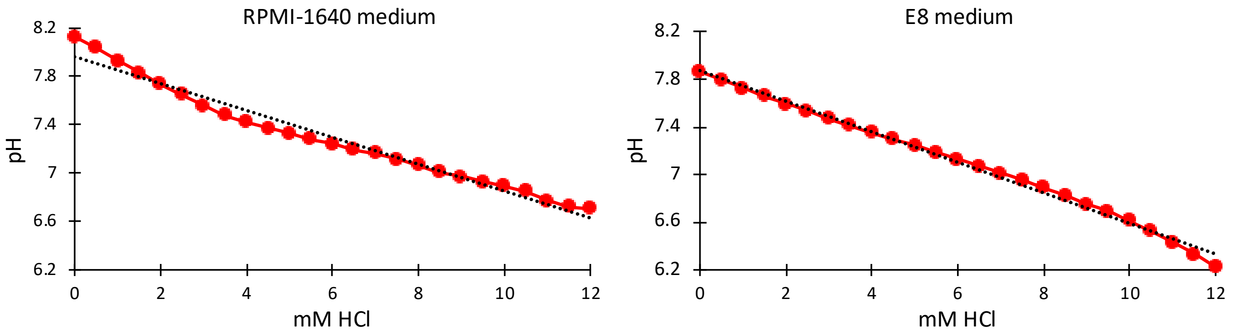

Figure S2. Measured pH buffering capacity of the RPMI-1640 and E8 medium. For RPMI-1640 and E8, the buffering capacity (β) was determined using a stepwise addition of 0.5mM HCl to the culture media and recorded pH at each stepwise addition.

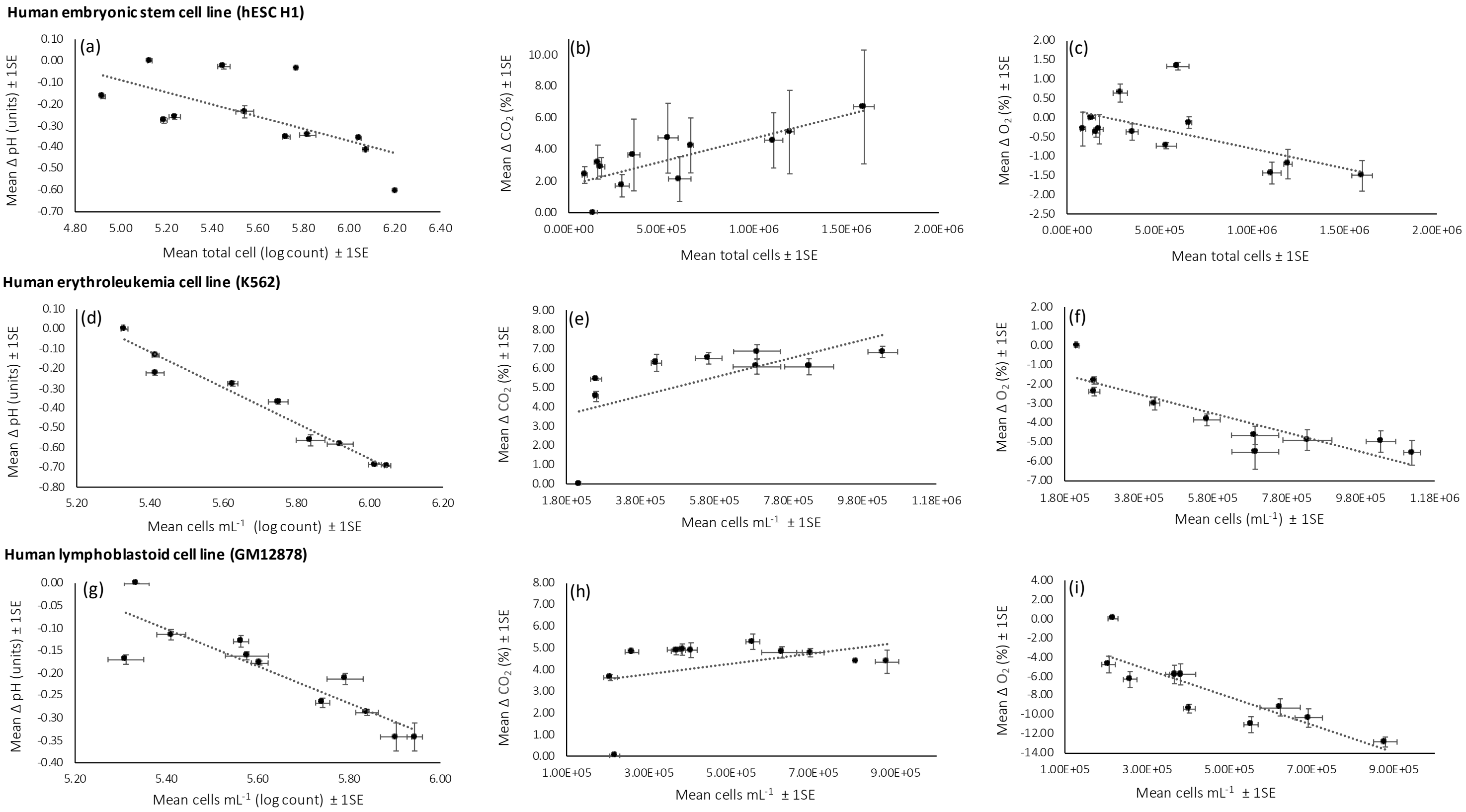

Figure S3. Relationships between changes in pH, dO_2, and dCO_2 and cell density in the H1 hESC (panels a, b, c), K562 (panels d, e, f), and GM12878 (panels g, h, i) cell line cultures. Delta (Δ) values for pH, dO_2, and dCO_2 represent the mean difference between the levels measured every eight hours during incubation minus measurements taken at time zero. Data points represent mean values across the three replicate flasks ± 1 standard error for each cell line.

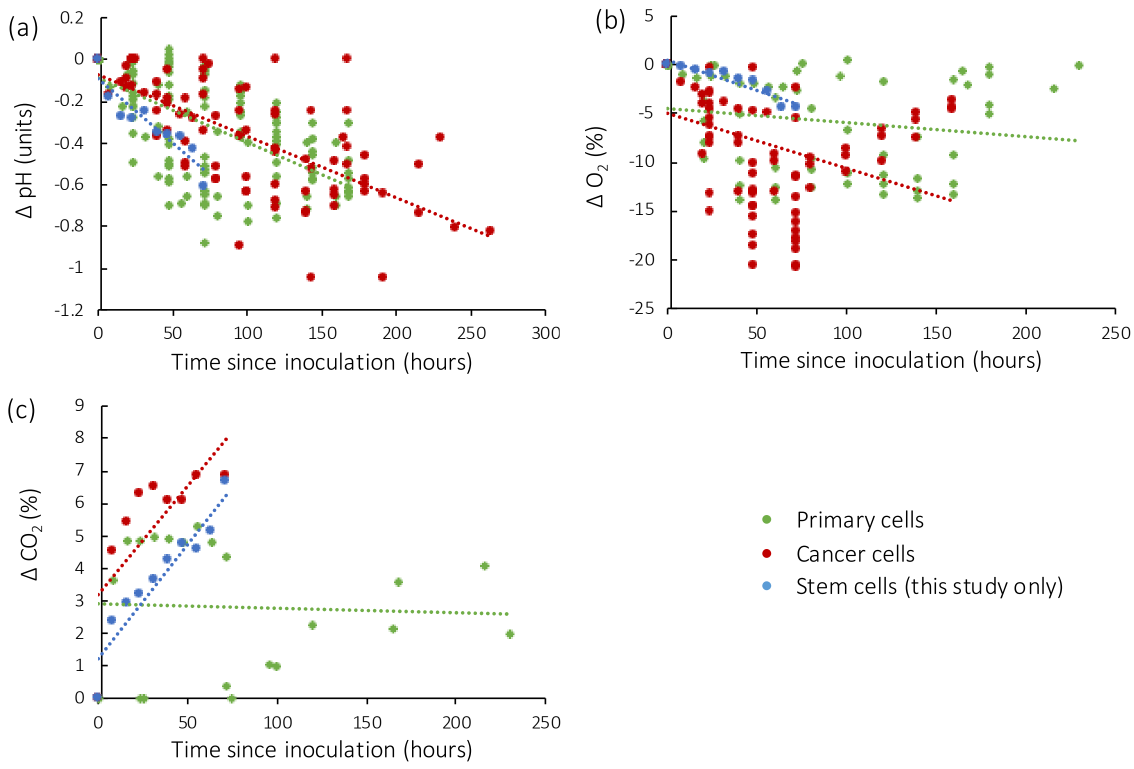

Figure S4. A comparison of relationships between changes in (a) pH, (b) dO_2, and (c) dCO_2 and incubation time (hours since inoculation) among primary cells, cancer cells, and stem cells (cf. Data Availability section). Delta (Δ) values for pH, dO_2, and dCO_2 represent the difference between the levels measured throughout the incubation periods minus measurements taken at time zeros. Data points represent raw values obtained from the published studies.

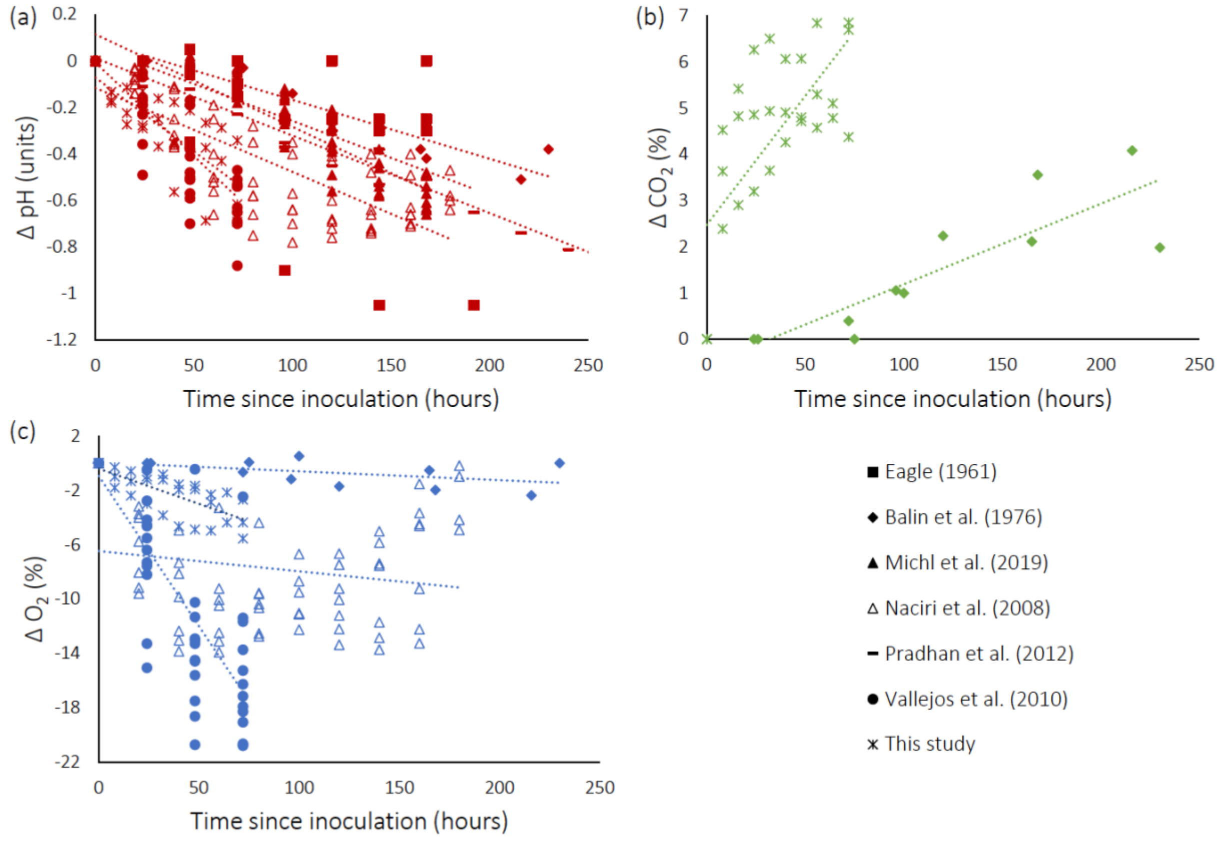

Figure S5. A comparison of relationships between changes in (a) pH, (b) dCO_2, and (c) dO_2 and incubation time (hours since inoculation) among the seven studies, regardless of cell type (cf. Data Availability section). Delta (Δ) values for pH, dO_2, and dCO_2 represent the difference between the levels measured throughout the incubation periods minus measurements taken at time zero. Data points represent raw values obtained from the published studies.

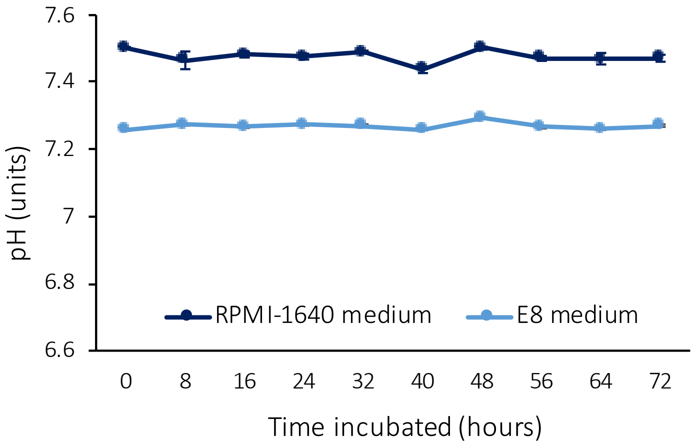

Figure S6 Time course of pH measured every eight hours in cell-free media (RPMI-1640 and E8 medium) during 72 hours of culture. Measurements conducted for three culture flasks per cell line (three biological replicates each). Data points represent mean ± 1 standard error.

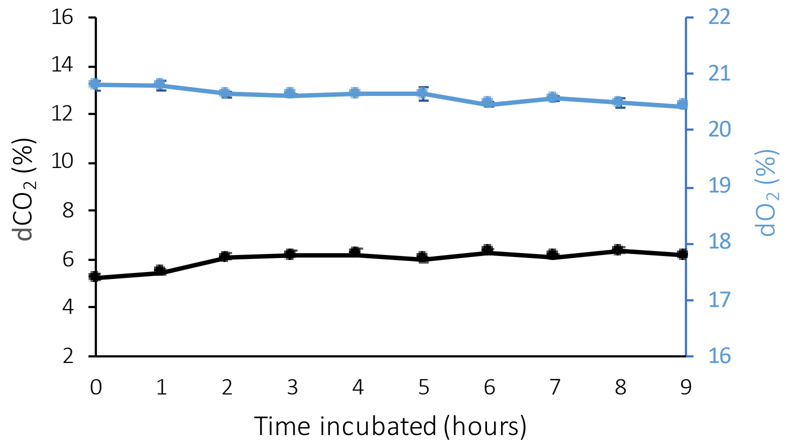

Figure S7 Temporally fine-scale measurements of dissolved gases show high stability of sensor spots. Time-course of dO_2 and dCO_2 measured hourly in RPMI-1640 medium in the absence of cells. Measurements conducted for three culture flasks per cell line (three biological replicates each). Data points represent mean ± 1 standard error.

Table S1. Summary of results for Pearson correlation analyses measuring the statistical association between changes in environmental parameters (O_2, CO_2, and pH) and cell growth (%) in cell line cultures of H1 hESC, K562, and GM12878.

Variables analyzed	Cell type	Pearson correlation value	Significance (p-value)	Number of observations	Figure location
**ΔO_2 – cell growth (%)**	H1 (hESCs)	-0.891	<0.001	12	Fig. 4a
	GM12878	-0.857	0.002	10	
	K562	-0.912	<0.001	9	
**ΔCO_2 - cell growth (%)**	H1 (hESCs)	0.808	0.001	12	Fig. 4b
	GM12878	0.413	0.235	10	
	K562	0.643	0.062	9	
**ΔpH - cell growth (%)**	H1 (hESCs)	-0.755	0.005	12	Fig. 4c
	GM12878	-0.880	<0.001	10	
	K562	-0.964	<0.001	9	

Table S2. Summary of results for Pearson correlation analyses measuring the statistical association between changes in environmental parameters (O_2, CO_2, and pH) in cell line cultures of H1 hESC, K562, and GM12878.

Variables analyzed	Cell type	Pearson correlation value	Significance (p-value)	Number of observations	Figure location
**ΔpH - ΔCO_2**	H1 (hESCs)	-0.582	< 0.001	36	Fig. 5a
	GM12878	-0.484	0.004	33	
	K562	-0.761	< 0.001	30	
**ΔO_2 - ΔCO_2**	H1 (hESCs)	-0.570	< 0.001	36	Fig. 5b
	GM12878	-0.574	< 0.001	33	
	K562	-0.734	< 0.001	30	
**ΔpH - ΔO_2**	H1 (hESCs)	0.824	< 0.001	36	Fig. 5c
	GM12878	0.920	< 0.001	33	
	K562	0.887	< 0.001	30	

Table S3. Summary of results for Pearson correlation analyses measuring the statistical association between changes in environmental parameters in mammalian cell cultures categorized as belonging to one of three main categories (primary cells, cancer cells, or stem cells).

**Variables analyzed**	**Cell type**	**Pearson correlation value**	**Significance (p-value)**	**Number of observations**	**Figure location**
**ΔpH - ΔCO_2**	Primary cells	-0.514	0.017	21	Fig. 6a
	Cancer cells	-0.768	0.016	9	
	Stem cells	-0.982	< 0.001	10	
**ΔO_2 - ΔCO_2**	Primary cells	-0.806	< 0.001	21	Fig. 6b
	Cancer cells	-0.868	0.002	9	
	Stem cells	-0.849	0.002	10	
**ΔpH - ΔO_2**	Primary cells	0.709	< 0.001	61	Fig. 6c
	Cancer cells	0.653	< 0.001	84	
	Stem cells	0.867	0.001	10	

Table S4. A detailed list of key resources used in the study, including reagents, equipment, software, and cell lines. The list specifies the manufacturer and manufacturer identifier for each resource used.

REAGENT or RESOURCE	SOURCE	IDENTIFIER
**Reagents**		
RPMI-1640	Thermo Fisher	31800105
FBS	Gibco	26140-079
PBS	Gibco	14190144
P/S	Gibco	15070-063
T-75 flasks	VWR	734-0050
Sodium bicarbonate	Gibco	25080-102
MycoAlert	Lonza	LT07-118
Buffer solution pH 4	Fisher Scientific	SB101-500
Buffer solution pH 7	Fisher Scientific	SB107-500
Buffer solution pH 10	Fisher Scientific	SB115-500
Essential 8 medium (E8)	ThermoFisher	A1517001
TrypLE	ThermoFisher	12604013
T-25 flask	VWR	10062-874
rhlaminin-521	Life Technologies	A29249
Lactate kit	Sigma	Mak064
**Equipment**		
Hera i150 incubator	Thermo Scientific	50116047
CO_2 meter	PreSens	CO2-1 SMA
O_2 meter	PreSens	OXY-2 SMA
CO_2 sensor dots	PreSens	SP-CD1-D5-rMy-US
Silicone glue	PreSens	SG-KiwikSil
O_2 sensor dots	PreSens	SP-PSt3-NAU-D10-YOP
Table top pH meter	Thermo Fisher	2115001
Countess II Automated cell counter	Thermo Fisher	AMQAF1000

Inverted microscope	Olympus	CKX53
**Synergy H1 plate reader**	BioTek	N/A
**<u>Software or platform</u>**		
SPSS	IBM	version 27
PreSens Measurement Studio 2	PreSens	Version 3.0.3
**<u>Cell lines</u>**		
GM12878	Coriell	N/A
K562	ATCC	N/A
H1 hESC	Wicell	N/A
