# Supplementary material for: In situ monitoring reveals cellular environmental instabilities in human pluripotent stem cell culture
Source: Commun Biol. 2022 Feb 8;5:119. doi: 10.1038/s42003-022-03065-w (PMC8826360; doi:10.1038/s42003-022-03065-w)
Supplement: Supplementary file 2 — Reporting Summary [file 42003_2022_3065_MOESM2_ESM.pdf]

nature research

Corresponding author(s): Carlos M. Duarte and Mo Li

Last updated by author(s): Dec 16, 2021

Reporting Summary

Nature Research wishes to improve the reproducibility of the work that we publish. This form provides structure for consistency and transparency in reporting. For further information on Nature Research policies, see our Editorial Policies and the Editorial Policy Checklist.

Statistics

For all statistical analyses, confirm that the following items are present in the figure legend, table legend, main text, or Methods section.

n/a	Confirmed	
[ ]	[x]	The exact sample size (*n*) for each experimental group/condition, given as a discrete number and unit of measurement
[ ]	[x]	A statement on whether measurements were taken from distinct samples or whether the same sample was measured repeatedly
[ ]	[x]	The statistical test(s) used AND whether they are one- or two-sided<br>*Only common tests should be described solely by name; describe more complex techniques in the Methods section.*
[x]	[ ]	A description of all covariates tested
[ ]	[x]	A description of any assumptions or corrections, such as tests of normality and adjustment for multiple comparisons
[ ]	[x]	A full description of the statistical parameters including central tendency (e.g. means) or other basic estimates (e.g. regression coefficient) AND variation (e.g. standard deviation) or associated estimates of uncertainty (e.g. confidence intervals)
[ ]	[x]	For null hypothesis testing, the test statistic (e.g. *F*, *t*, *r*) with confidence intervals, effect sizes, degrees of freedom and *P* value noted<br>*Give P values as exact values whenever suitable.*
[x]	[ ]	For Bayesian analysis, information on the choice of priors and Markov chain Monte Carlo settings
[x]	[ ]	For hierarchical and complex designs, identification of the appropriate level for tests and full reporting of outcomes
[ ]	[x]	Estimates of effect sizes (e.g. Cohen's *d*, Pearson's *r*), indicating how they were calculated

Our web collection on statistics for biologists contains articles on many of the points above.

Software and code

Policy information about availability of computer code

Data collection: The commercial software SPSS (version 27) was used in all statistical analyses.

Data analysis: The type of analyses (in SPSS version 27 used are stated in the text of the study.

For manuscripts utilizing custom algorithms or software that are central to the research but not yet described in published literature, software must be made available to editors and reviewers. We strongly encourage code deposition in a community repository (e.g. GitHub). See the Nature Research guidelines for submitting code & software for further information.

Data

Policy information about availability of data

All manuscripts must include a data availability statement. This statement should provide the following information, where applicable:

- Accession codes, unique identifiers, or web links for publicly available datasets
- A list of figures that have associated raw data
- A description of any restrictions on data availability

The dataset containing published data of environmental parameters measured in routine cell line cultures generated and analyzed during the current study is available at https://datadryad.org/stash/share/nsp3ZpSdb9nMewd6sDQ8ahjKy91yBbjD5pNLD39uA48.

Field-specific reporting

Life sciences study design

All studies must disclose on these points even when the disclosure is negative.

Sample size	The sample size of published data was determined by availability. We, therefore, extracted and analyzed all available data for this component of the study. For the experimental component, we chose a sample size that was equivalent to power of previous studies that conducted similar experiments. The sample size was chosen as a balance between replication and logistic feasibility.
Data exclusions	In the publication search, we used a combination of keywords in PubMed NCBI® and Google Scholar search engines, which included “cell culture” AND “pH”, “O2”, “CO2”, “buffering capacity”, “dissolved oxygen”, “dissolved carbon dioxide”, “dissolved gases”. We extracted mean pH, O2 and CO2 values, as well as the time at which the values were recorded throughout the incubation (reported in hours since inoculation). We used these data to produce Δ values that represented the difference between measurements taken at time zero minus the levels measured at intervals during the cultures. We collected mean pH, dO2 and dCO2 values for all cell cultures within each publication, including those of the same cell type with slight differences in media formulations (e.g., higher or lower concentrations of serum or differing concentrations of buffers) to represent the diversity of changes expected in mammalian cell cultures.
Replication	We undertook several experiments (with the same replication) to ensure the accuracy and precision of our measurements. Each repeated experiment showed the same trends, but the final experimental data presented had the highest precision and accuracy of environmental measurements, possible.
Randomization	All culture flasks were randomly allocated to each environmental parameter. Each flask was also randomly sampled for each time point.
Blinding	N/A

Reporting for specific materials, systems and methods

We require information from authors about some types of materials, experimental systems and methods used in many studies. Here, indicate whether each material, system or method listed is relevant to your study. If you are not sure if a list item applies to your research, read the appropriate section before selecting a response.

Materials & experimental systems

n/a	Involved in the study
☒	☐ Antibodies
☐	☒ Eukaryotic cell lines
☒	☐ Palaeontology and archaeology
☒	☐ Animals and other organisms
☒	☐ Human research participants
☒	☐ Clinical data
☒	☐ Dual use research of concern

Methods

n/a	Involved in the study
☒	☐ ChIP-seq
☒	☐ Flow cytometry
☒	☐ MRI-based neuroimaging

Eukaryotic cell lines

Policy information about cell lines

Cell line source(s)	The human pluripotent stem cells (H1) was obtained from WiCell. The human lymphoblastoid cell line GM12878 was obtained from Coriell institute. The human erythroleukemia cell line K562 was obtained from ATCC.
Authentication	These cell lines were obtained from reliable commercial sources.
Mycoplasma contamination	We routinely check for mycoplasma contamination using lonza mycoalert kit, as described in the methods of the paper.
Commonly misidentified lines (See ICLAC register)	N/A
